# HPV33 DNA methylation measurement improves cervical pre-cancer risk estimation of an HPV16, HPV18, HPV31 and \textit{EPB41L3} methylation classifier

**DOI:** 10.3233/CBM-150507

**Published:** 2015

**Authors:** Adam R. Brentnall, Natasa Vasiljevic, Dorota Scibior-Bentkowska, Louise Cadman, Janet Austin, Jack Cuzick, Attila T. Lorincz

**Affiliations:** Centre for Cancer Prevention, Wolfson Institute of Preventive Medicine, Barts and The London School of Medicine, Queen Mary University of London, London, UK

**Keywords:** Cervical intraepithelial neoplasia, DNA methylation, early detection of cancer, human papillomavirus 33, human papillomavirus DNA tests, uterine cervical neoplasms

## Abstract

**BACKGROUND:** Persistent infection %by with high risk human papillomavirus (hrHPV) types causes cervical cancer but most women who test positive are at very low risk of neoplasia. Strategies are needed which can retain high sensitivity of hrHPV testing but reduce the number of false-positives. We showed previously that a combination DNA methylation triage assay for HPV types 16, 18 and 31 and human gene *EPB41L3* was useful to identify high grade cervical lesions.

**OBJECTIVE:** Assess whether measurement of DNA methylation in HPV type 33 can improve the previous classifier.

**METHODS:** A London colposcopy referral group of 1493 women of whom 556 (37%) had histologically-confirmed CIN (cervical intraepithelial neoplasia) 2 or 3 that included 114 HPV33 positive women with methylation measured for three L2 CpGs 5557, 5560 and 5566. Discrimination performance was assessed for the new classifier S5, built by adding HPV33 to the earlier classifier.

**RESULTS:** HPV33 methylation measurement improved prediction among HPV33 positive women. Receiver operating characteristic analyses showed an area under the curve (AUC) for HPV33 methylation of 0.68 (95% CI 0.57-0.78). The earlier risk score was significantly improved by HPV33 methytlation (AUC = 0.82 vs 0.80; P < 0.001). For 90% sensitivity the specificity for CIN2/3 was 49% (95% CI 46-52%). **CONCLUSIONS:** Measurement of HPV33 DNA methylation contributes independent diagnostic information to *EPB41L3* and HPV16, HPV18 and HPV31, and is superior to genotyping. Other HPV and human methylation target regions might be useful to further improve S5.


Cancer Biomarkers 15 (2015) 669–675
DOI 10.3233/CBM-150507
IOS Press

### HPV33 DNA methylation measurement improves cervical pre-cancer risk estimation of an HPV16, HPV18, HPV31 and *EPB41L3* methylation classifier

Adam R. Brentnall, Natasa Vasiljevic, Dorota Scibior-Bentkowska, Louise Cadman, Janet Austin, Jack Cuzick and Attila T. Lorincz*
*Centre for Cancer Prevention, Wolfson Institute of Preventive Medicine, Barts and The London School of Medicine, Queen Mary University of London, London, UK*

**Abstract.**
**BACKGROUND:** Persistent infection with high risk human papillomavirus (hrHPV) types causes cervical cancer but most women who test positive are at very low risk of neoplasia. Strategies are needed which can retain high sensitivity of hrHPV testing but reduce the number of false-positives. We showed previously that a combination DNA methylation triage assay for HPV types 16, 18 and 31 and human gene *EPB41L3* was useful to identify high grade cervical lesions.
**OBJECTIVE:** Assess whether measurement of DNA methylation in HPV type 33 can improve the previous classifier.
**METHODS:** A London colposcopy referral group of 1493 women of whom 556 (37%) had histologically-confirmed CIN (cervical intraepithelial neoplasia) 2 or 3 that included 114 HPV33 positive women with methylation measured for three L2 CpGs 5557, 5560 and 5566. Discrimination performance was assessed for the new classifier S5, built by adding HPV33 to the earlier classifier.
**RESULTS:** HPV33 methylation measurement improved prediction among HPV33 positive women. Receiver operating characteristic analyses showed an area under the curve (AUC) for HPV33 methylation of 0.68 (95% CI 0.57–0.78). The earlier risk score was significantly improved by HPV33 methytlation (AUC = 0.82 vs 0.80; $P < 0.001$). For 90% sensitivity the specificity for CIN2/3 was 49% (95% CI 46–52%).
**CONCLUSIONS:** Measurement of HPV33 DNA methylation contributes independent diagnostic information to *EPB41L3* and HPV16, HPV18 and HPV31, and is superior to genotyping. Other HPV and human methylation target regions might be useful to further improve S5.

Keywords: Cervical intraepithelial neoplasia, DNA methylation, early detection of cancer, human papillomavirus 33, human papillomavirus DNA tests, uterine cervical neoplasms

### 1. Introduction

Pap smear cytological screening has helped save many womens' lives [15] despite having only modest sensitivity for cervical neoplasia and pre-cancerous lesions. Many studies have reported that cytology misses more women with high grade cervical intraepthelial neoplasia (CIN 2/3), for whom treatment may prevent cervical cancer, than testing for high risk human papilloma virus (hrHPV) types (e.g. [4]). However, improving on an established, effective and specific cytology-based screening strategy is not straightforward [2]. Most HPV infections clear and so HPV testing also detects many more women who are not at risk for CIN2/3. This is part of the reason that in 2011 some organisations in the USA recommended

*Corresponding author: Attila T. Lorincz, Centre for Cancer Prevention, Wolfson Institute of Preventive Medicine, Barts and The London School of Medicine, Queen Mary University of London, Charterhouse Square, London EC1M 6BQ, UK. Tel.: +44 20 7882 3540; Fax: +44 20 7882 3890; E-mail: a.lorincz@qmul.ac.uk.

ISSN 1574-0153/15/$35.00 © 2015 – IOS Press and the authors. All rights reserved
This article is published online with Open Access and distributed under the terms of the Creative Commons Attribution 4.0 International License.

cervical screening based on cytology in combination with hrHPV testing [7]. More recent evidence has suggested that hrHPV testing alone might be used as a primary screening test, with cytology reserved as a triage test [21]. An alternative or addition to cytology triage is a molecular test such as genotyping for HPV16 or HPV18 that can be performed reflexively from the original HPV screening specimens. Unfortunately the positive predictive value of HPV16 and especially of HPV18 infections is quite low, thus the development of an improved molecular triage test for hrHPV-positive women is of potentially huge practical value.

Our long-term objective is to develop a practical molecular triage test that maintains the high sensitivity of 14-group cocktail hrHPV testing but with far fewer false positives, thereby allowing a fully molecular triage test. This would have obvious clinical utility in new automated HPV-based strategies for cervical screening. An area that has shown some promise for this objective is the quantitative measurement of DNA methylation. These epigenetic differences can be accurately measured by molecular methods, and are linked with the development of a variety of cancers [10]. We and other research groups have observed that measurement of differential methylation of certain HPV CpG sites is useful for stratifying HPV type-specific risk [8,11–13,19]. Additionally, methylation of CpGs in the promoters or introns of human genes has shown some value [5,6,9,14,20]. In earlier work we demonstrated that combining the measurement of human and HPV methylation might produce a better classifier than either alone [1]. A risk score was developed using DNA methylation measurement in a group of women attending for coloposcopy in London, by selecting risk factors from a large number of potential CpG sites. The CpGs selected were then assayed on a later cohort from the same population, for validation and model updating. This resulted in a risk score (S4) using DNA methylation in a human gene *EPB41L3*, and selected CpGs in L1 and L2 regions of HPV16, HPV18 and HPV31. Here, we consider further methylation assays of HPV33 with an aim to improve S4, and take another step towards a practical molecular test for triage of hrHPV women in cervical cancer screening.

### 2. Materials and methods

### 2.1. Objective

The data that we used in [1] were subsequently extended, by the assessment of 27 CpG sites in HPV33 positive women within the same cohorts [19]. The present article focuses on whether HPV33 DNA methylation testing might add independent triage information to the previously developed S4 risk score. That is, the study aims to assess the information HPV33 methylation levels might add to that from 5 HPV16 CpG sites in L1 and L2 regions, 6 HPV18 L2 sites, 2 HPV31 L1 sites, and 3 CpGs in the human gene *EPB41L3*.

### 2.2. Patients

Data from 1493 hrHPV positive women from the Predictors 1 (P1) and 2 (P2) studies at St. Mary's and Hammersmith Hospitals in London were used to assess the additional value of HPV33 DNA methylation, with a focus on increased methylation indicating increasing risk for CIN2/3. The women had been referred to colposcopy because of an abnormal screening cytology result (persistent borderline or mild, moderate or severe dyskaryosis; equivalent to a diagnosis of atypical squamous cells of undetermined significance or worse in the United States of America (USA)). All women underwent coloposcopic examination, with biopsy and treatment as appropriate. The studies were approved by the local research ethics committees and all women analysed provided written consent and the study conforms with The Code of Ethics of the World Medical Association (Declaration of Helsinki), printed in the British Medical Journal (18 July 1964); full details are available elsewhere [17,18].

### 2.3. Specimen characteristics

Cervical samples were taken prior to colposcopy by a Cervex brush and placed into PreservCyt (Hologic, Danbury, USA) and stored at −70°C until the DNA methylation assays were run. Details of the DNA extraction and conversion are as previously described [1].

### 2.4. Assay methods

From the complete P1 and P2 groups, 126 HPV33 samples were selected based on genotyping by the Linear Array (Roche Molecular Systems, Pleasanton, USA) and a qPCR test in P1, and the BD HPV test (BD Diagnostics, Burlington, USA) in P2. The tests are functionally equivalent to other hrHPV tests [18].

Methylation assays were based on PCR and quantitative pyrosequencing as previously described [1,19]. Primers for 7 PCRs in L2, L1 and URR covering 27

CpG positions in HPV33 were obtained using PyroMark Assay Design software version 2.0.1.15 (Qiagen, Venlo Netherlands), as given in Supplemental Table 3 of [19]. Primers covered dense CpG areas in a single amplicon of less than 300bp and did not overlap any CpG dyads. The internal control for total bisulfite conversion was a non-CpG cytosine in the region for pyrosequencing.

PCR and pyrosequencing were performed as previously described [1]. All 27 CpG positions failed for 9/126 samples that were tested for HPV33 methylation, these were treated as HPV33 negative.

### 2.5. *Primary endpoint and predictor variables*

The same subset of hrHPV referral samples from the P1 (between 2005 and 2007) and P2 (2007–2009) studies were analysed as used to develop the S4 score based on HPV16, HPV18, HPV31 and *EPB41L3* [1]. This included 114/117 samples from P1 and P2 with HPV33 methylation data. The primary endpoint was histologically-confirmed CIN2/3, taking the highest grade of abnormality seen in the punch or treatment biopsy specimen. Histopathology was first reported locally and then centrally reviewed.

Details of the performance and correlation of individual HPV33 CpGs were reported earlier [19]. The analysis identified 6 CpGs in L2 and 3 CpGs in the L1 regions as being associated with CIN status. For the present analysis we considered the group of the best performing CpGs in L2 that may be assessed with a single primer. These were HPV33 CpGs at nucleotide positions 5557, 5560, 5566. Mean methylation at these positions was used as the main predictor variable where zero methylation was imputed if the measurement failed. For one sample the measurement failed at all three sites, another one failed at two sites and one sample failed at one site. The main HPV33 predictor was examined alongside S4, which used the mean methylation of the CpGs within a gene or HPV types at nucleotides: *EPB41L3*: 438, 427, 425; HPV16-L1: 6367, 6389; HPV18-L2: 4256, 4261, 4265, 4269, 4275, 4281; and HPV31-L1: 6352 and 6364 and the proportion of CpGs methylated in HPV16-L2 sites: 4238, 4259, 4275.

### 2.6. *Statistical analysis methods*

Spearman correlation was calculated between HPV33 methylation and S4. Likelihood ratio chi-squared statistics from a logistic regression model with terms for S4, HPV33 positivity and HPV33 methylation were used to assess the benefit of HPV33 methylation. A new rule called Score 5 (S5) was developed from a logistic regression model with one unknown parameter for HPV33 and S4 as an offset. The S5 coefficients were rescaled so that the score theoretically ranged between 0 and 100. Discrimination was assessed using receiver operating characteristic (ROC) plots, and the area under the curve (AUC). A sensitivity analysis was undertaken to assess whether only using methylation from one HPV type (with preference to (i) HPV16, (ii) HPV33 and (iii) HPV31) was more predictive than our model assumption of treating multiple infections independently. Comparisons were made to an HPV genotype rule that ordered risk by positive predictive value in the sample (respectively HPV33, HPV16, HPV31 and HPV18, as [3]).

Differences between methylation distributions by CIN status were tested by the Mann-Whitney test. DeLong confidence intervals were used for AUC statistics, Wilson confidence intervals for binary outcomes and profile likelihood for logistic regressions. All $P$-values were two-sided. Analysis was undertaken by using the statistical software GNU R 2.15.1 [16].

Table 1
Summary statistics for mean methylation at HPV33 CpGs 5557, 5560, 5566

| Statistic | HPV33 |
|---|---|
| *Spearman ρ* | |
| S4 (all samples) | 0.28 ($P = 0.003$) |
| S4 (subset: HPV16,18,31+) | 0.22 ($P = 0.216$) |
| *HPV33 + Methylation* | |
| Mean (IQR), <CIN2/3 | 10% (4–13) |
| Mean (IQR), CIN2/3 | 16% (8–21) |
| $P$ | 0.001 |
| *When added to S4* | |
| Change LR-$\chi^2$ | 50.2 |
| Genotype | 43.8 ($P < 0.001$) |
| Methylation | 6.4 ($P = 0.012$) |
| Increase AUC | 0.02 ($P < 0.001$) |

### 3. Results

Of 114 HPV33 positive women in the cohort, 71 (62%) had CIN2/3. This HPV33 positive group was at elevated risk compared with the cohort, where 556/1493 (37%) had CIN2/3.

Summary statistics are presented in Table 1. HPV33 added significantly to S4 ($P < 0.001$), where DNA methylation provided additional information to the

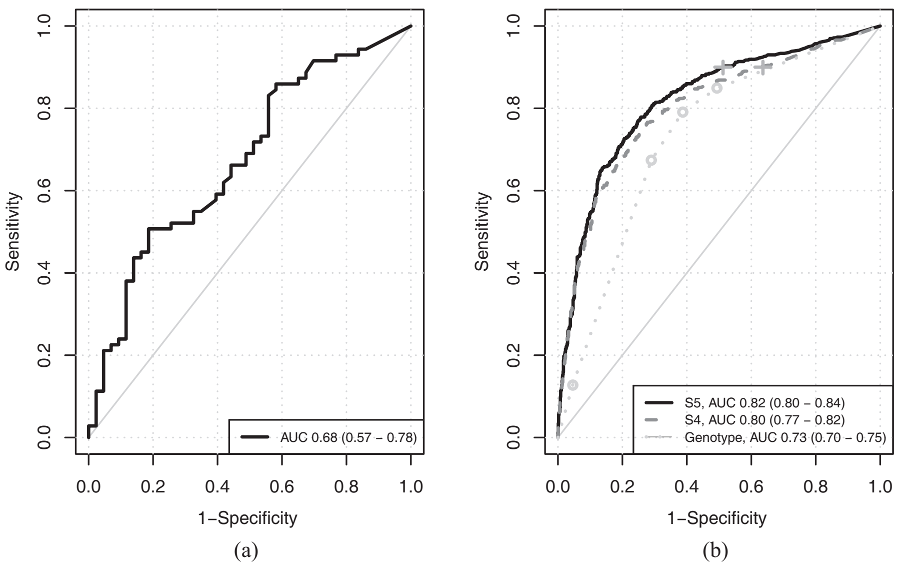

Fig. 1. ROC plots: (a) HPV33 positive samples and mean HPV33 methylation, (b) all samples and S4, S5, and HPV genotype methylation positivity ordered by positive predictive value, showing as incremental points (light gray circles) starting at the origin: HPV negative, HPV33, HPV16, HPV31 and HPV18 respectively. Selected 95% confidence regions at 90% sensitivity cutpoints are also shown (grey +). The difference between S4 and S5 was significant ($P < 0.001$).

HPV33 genotype (LR-$\chi^2$ 6.4, $P = 0.012$), and Fig. 1(a) shows how it helped to rank order the risk of HPV33 positive women (AUC 0.68, 95% CI 0.57–0.78). Mean HPV33 methylation was moderately correlated with the earlier risk score S4 (Spearman 0.28).

A new risk score S5 (score with 1 human gene and 4 HPV types) was estimated as

$$S5 = 30.9 \times \textit{EPB41L3} + 13.7 \times \text{HPV16-L1}$$

$$+4.3 \times \text{HPV16-L2} + 8.4 \times \text{HPV18-L2}$$

$$+22.4 \times \text{HPV31-L1} + 20.3 \times \text{HPV33-L1}$$

where HPV33-L2 is the mean of the CpG sites (5557, 5560, 5566) and the other S4 terms were described above in statistical methods. A sensitivity analysis supported treating multiple infections independently. A model that treated HPV types independently fitted the data much better than one where only the highest-risk type was used (LR-$\chi^2 = 443.0$ vs. 453.9). This suggested that, for example, when a woman has both HPV33 and HPV16, it is better for risk prediction to use the methylation levels in both, than only HPV16.

S4 and S5 were substantially better for risk stratification than genotyping alone (Fig. 1(b)). HPV33 methylation in S5 improved performance over S4 at the high sensitivity end of the ROC, and because the S4 components were fixed as previously, the improvements were only due to different predictions for HPV33 positive women. Figure 2 shows the change in scores, where most of the women had an increased risk with S5.

The performance of S5 at the 90% sensitivity point is summarised in Table 2, at this cutpoint specificity was 49% (95% CI 46–52%) which is superior to the corresponding specificity of 36% (33–40%) achieved by S4. A subgroup analysis separating HPV infections included in the S5 classifier from other high risk HPV types showed that on the basis of *EPB41L3* alone, sensitivity of 49% and specificity of 75% were reached.

### 4. Discussion

We developed a new risk classifier S5 by expanding a previously described S4 classifier to include DNA methylation data from three selected sites in HPV33 L2. The classifier improved the ability to identify high-grade disease. In the hrHPV positive subgroup with sensitivity set at 90% for CIN2/3, the specificity of S5 was 49% (95% CI 46–52%). In comparison, the earlier risk score S4 had a specificity of 36% (33–40%), and the overall improvement in AUC from 0.80 to 0.82 was highly significant [23] (Fig. 1 and Table 1). A subgroup analysis showed that among the women infected with other hrHPV types than those included in S5 classifier, only 15% were diagnosed with CIN2/3 and the S5 classifier was able to identify 49% of these. The lower sensitivity in this subgroup, where detec-

Table 2
Specificity and positive predictive value (95% CI) from S5 at 90% sensitivity (cutpoint is 0.8)

| Group | n | CIN2/3 (%) | Sensitivity | Specificity | PPV |
|---|---|---|---|---|---|
| All samples | 1493 | 556 (37%) | 90% (87–92) | 49% (46–52) | 51% (49–54) |
| HPV16,18,31,33 | 935 | 472 (50%) | 97% (96–99) | 21% (18–25) | 56% (53–59) |
| Other hrHPV | 558 | 84 (15%) | 49% (38–59) | 75% (71–79) | 26% (22–30) |

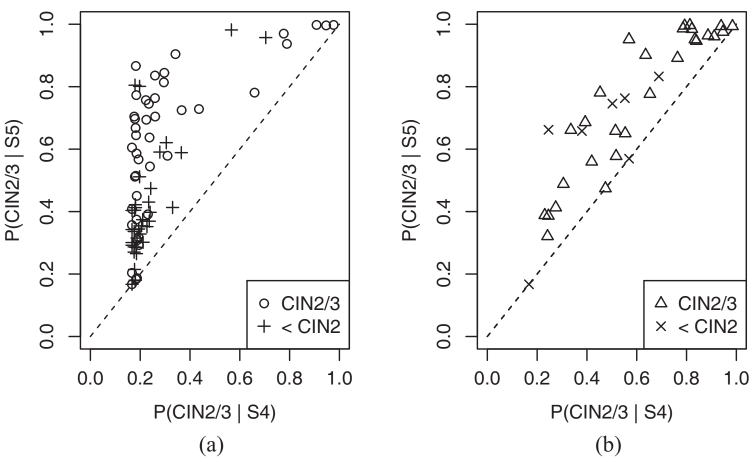

Fig. 2. Change in predicted risk for HPV33 positive women for (a) single HPV33 infections and (b) multiple infections with HPV33 and any other hrHPV type with methylation data (HPV16 or HPV18 or HPV31). The scores have been normalised to show the fitted probability of CIN2/3 for each HPV33 positive woman in the sample.

tion is based on methylation of *EPB41L3* alone, suggested that addition of methylation assays for other hrHPV may be warranted. Among the 26 human genes originally considered for the human gene methylation component of the S4 classifier [22], *EPB41L3* showed the strongest potential. However, the gene selection was based on earlier reports either describing promising QMSP assays or showing aberrant methylation by at least two different research groups. More comprehensive ways of identifying novel methylation targets, such as methylation genome sequencing, are likely to provide additional human genes that can improve current methylation-based classifiers.

HPV33 methylation improved risk stratification within the HPV33 positive group (Fig. 2) and added independent information to S4, beyond genotype information. HPV33 contributed more than twice the information of HPV18 to risk evaluation (Supplemental Table S1), and as demonstrated in Fig. 1(b) S5 was a much stronger risk predictor (AUC = 0.82) than genotyping alone (AUC = 0.73, $P < 0.001$). Our evidence indicates that that the incremental improvement from DNA methylation of HPV33 is a real step towards a clinically useful triage risk score, which is particularly relevant at a time when hrHPV testing is likely to become the primary screening test [21].

A limitation of our study is that the CpG sites were selected and evaluated in the same data, and the sample had a relatively high CIN2/3 prevalence. Thus further validation in an independent test set is needed, particularly in primary screening populations. However, the study is relatively large, and part of the reason for fixing the S4 components to be as derived earlier [1] was to help avoid overfitting. Another limitation is that the specificity attained for a fixed 90% sensitivity in a screening population might be different since all the women in our sample had abnormal cytology. Future work is planned to assess the performance of S4 and S5 and appropriate cutpoints for women in a screening population with a preponderance of normal cytology; these women would be detected through primary hrHPV screening but were not represented in our cohort.

In conclusion, quantitative DNA methylation assays based on a human gene and hrHPV types show good promise for the development of molecular tests to triage hrHPV women to colposcopy in cervical cancer screening programs. Furthermore, the S5 classifier is suggested as a possible reflex test to hrHPV screening not as a replacement. We continue to search for additional biomarkers that may further improve the sensitivity and specificity of DNA methylation classifiers and ultimately design a methylation based test that could be used in primary screening.

### Acknowledgments

The authors thank all the women who enrolled in the Predictors 1 and 2 studies. We thank Deirdre Lyons for her work as the Consultant in the Predictors 1 and 2 studies at St. Mary's hospital.

### Conflict of interest

Dr. Cuzick reports grants, personal fees and non-financial support from Abbott, Beckton Dickinson, Hologic, Qiagen, Genera and Roche, grants and non-financial support from Trovagene, and personal fees and non-financial support from Cepheid during the conduct of the study. All the other authors report no conflict of interests.

### Funding

This work was funded by Cancer Research UK [Grant number C569/A16891].

### Ethical approval

Hammersmith and Queen Charlottes & Chelsea Research Ethics Committee REC no 05/Q0406/57.

### References

[1] A.R. Brentnall, N. Vasiljević, D. Scibior-Bentkowska, L. Cadman, J. Austin, A. Szarewski, J. Cuzick and A.T. Lorincz. A DNA methylation classifier of cervical precancer based on human papillomavirus and human genes, *Int J Cancer* **135** (2014), 1425–1432.

[2] T.T. Cox, P.E. Castle, C.M. Behrens, A. Sharma, T.C. Wright, J. Cuzick and Athena HPV Study Group, Comparison of cervical cancer screening strategies incorporating different combinations of cytology, HPV testing, and genotyping for HPV 16/18: results from the ATHENA HPV study, *American Journal of Obstetrics and Gynecology* **208** (2013), 184.e1–184.e11.

[3] J. Cuzick, L. Ho, G. Terry, M. Kleeman, M. Giddings, J. Austin, L. Cadman, L. Ashdown-Barr, M.J. Costa and A. Szarewski, Individual detection of 14 high risk human papilloma virus genotypes by the PapType test for the prediction of high grade cervical lesions, *Journal of Clinical Virology* **60** (2014), 44–49.

[4] J. Cuzick, A. Szarewski, G. Terry, L. Ho, A. Hanby, P. Maddox, M. Anderson, G. Kocjan, S.T. Steele and J. Guillebaud, Human papillomavirus testing in primary cervical screening, *Lancel* **345** (1995), 1533–1536.

[5] J.J. Eijsink, A. Lendvai, V. Deregowski, H.G. Klip, G. Verpooten, L. Dehaspe, G.H. de Bock, H. Hollema, W. van Criekinge, E. Schuuring, A.G. van der Zee and G.B. Wisman, A four-gene methylation marker panel as triage test in high-risk human papillomavirus positive patients, *Int J Cancer* **130** (2012), 1861–1869.

[6] A.T. Hesselink, D.A.M. Heideman, R.D.M. Steenbergen, M. Gök, F.J. van Kemenade, S.M. Wilting, J. Berkhof, C.J.L.M. Meijer and P.J.F. Snijders, Methylation marker analysis of self-sampled cervico-vaginal lavage specimens to triage high-risk HPV-positive women for colposcopy, *Int J Cancer* **135** (2014), 880–886.

[7] W.K. Huh, K.A. Ault, D. Chelmow, D.D. Davey, R.A. Goulart, F.A. Garcia, W.K. Kinney, S.S. Massad, E.J. Mayeaux, D. Saslow, M. Schiffman, N. Wentzensen, H.W. Lawson and M.H. Einstein, Use of primary High-Risk human papillomavirus testing for cervical cancer screening: Interim clinical guidance, *Obstetrics and Gynecology* **136** (2015), 178–82.

[8] S. Jain, T.K. Wojdacz and Y.-H.H. Su, Challenges for the application of DNA methylation biomarkers in molecular diagnostic testing for cancer, *Expert Review of Molecular Diagnostics* **13** (2013), 283–294.

[9] H.-C.C. Lai, Y.-W.W. Lin, R.-L.L. Huang, M.-T.T. Chung, H.-C.C. Wang, Y.-P.P. Liao, P.-H.H. Su, Y.-L.L. Liu and M.-H.H. Yu, Quantitative DNA methylation analysis detects cervical intraepithelial neoplasms type 3 and worse, *Cancer* **116** (2010), 4266–4274.

[10] A.T. Lorincz, Cancer diagnostic classifiers based on quantitative DNA methylation, *Expert Review of Molecular Diagnostics* **14** (2014), 293–305.

[11] A.T. Lorincz, A.R. Brentnall, N. Vasiljević, D. Scibior-Bentkowska, A. Castanon, A. Fiander, N. Powell, A. Tristram, J. Cuzick and P. Sasieni, HPV16 l1 and l2 DNA methylation predicts high-grade cervical intraepithelial neoplasia in women with mildly abnormal cervical cytology, *Int J Cancer* **133** (2013), 637–644.

[12] L. Mirabello, M. Schiffman, A. Ghosh, A.C. Rodriguez, N. Vasiljevic, N. Wentzensen, R. Herrero, A. Hildesheim, S. Wacholder, D. Scibior-Bentkowska, R.D. Burk and A.T. Lorincz, Elevated methylation of HPV16 DNA is associated with the development of high grade cervical intraepithelial neoplasia, *Int J Cancer* **132** (2013), 1412–1422.

[13] L. Mirabello, C. Sun, A. Ghosh, A.C. Rodriguez, M. Schiffman, N. Wentzensen, A. Hildesheim, R. Herrero, S. Wacholder, A. Lorincz and R.D. Burk, Methylation of human papillomavirus type 16 genome and risk of cervical precancer in a costa rican population, *Journal of the National Cancer Institute* **104** (2012), 556–565.

[14] R.M. Overmeer, J.A. Louwers, C.J. Meijer, F.J. van Kemenade, A.T. Hesselink, N. Fransen, S.M. Wilting, D.A. Heideman, R.H. Verheijen, A. Zaal, M.M. van Baal, J. Berkhof, P.J. Snijders and R.D. Steenbergen, Combined CADM1 and MAL promoter methylation analysis to detect (pre-)malignant cervical lesions in high-risk HPV-positive women, *Int J Cancer* **129**, 2218–2225.

[15] J. Peto, C. Gilham, O. Fletcher and F.E. Matthews, The cervical cancer epidemic that screening has prevented in the UK, *Lancet* **364** (2004), 249–256.

[16] R Core Team, *R: A Language and Environment for Statistical*

*Computing*, R Foundation for Statistical Computing, Vienna, Austria, 2012, ISBN 3-900051-07-0.

[17] A. Szarewski, L. Ambroisine, L. Cadman, J. Austin, L. Ho, G. Terry, S. Liddle, R. Dina, J. McCarthy, H. Buckley, C. Bergeron, P. Soutter, D. Lyons and J. Cuzick, Comparison of predictors for High-Grade cervical intraepithelial neoplasia in women with abnormal smears, *Cancer Epidemiology Biomarkers & Prevention* **17** (2008), 3033–3042.

[18] A. Szarewski, D. Mesher, L. Cadman, J. Austin, L. Ashdown-Barr, L. Ho, G. Terry, S. Liddle, M. Young, M. Stoler, J. McCarthy, C. Wright, C. Bergeron, W.P. Soutter, D. Lyons and J. Cuzick, A comparison of seven tests for high grade cervical intraepithelial neoplasia in women with abnormal smears: the predictors 2 study, *Journal of Clinical Microbiology* **50** (2012), 1867–1873.

[19] N. Vasiljević, D. Scibior-Bentkowska, A. Brentnall, J. Cuzick and A. Lorincz, A comparison of methylation levels in HPV18, HPV31 and HPV33 genomes reveals similar associations with cervical precancers, *Journal of Clinical Virology* **59** (2014), 161–166.

[20] V.M. Verhoef, D.A. Heideman, F.J. van Kemenade, L. Rozendaal, R.P. Bosgraaf, A.T. Hesselink, R.L. Bekkers, L.F. Massuger, R.D. Steenbergen, P.J. Snijders, J. Berkhof and C.J. Meijer, Methylation marker analysis and HPV16/18 genotyping in high-risk HPV positive self-sampled specimens to identify women with high grade CIN or cervical cancer, *Gynecologic Oncology* **135** (2014), 58–63.

[21] T.C. Wright, M.H. Stoler, C.M. Behrens, A. Sharma, G. Zhang and T.L. Wright, Primary cervical cancer screening with human papillomavirus: End of study results from the ATHENA study using HPV as the first-line screening test, *Gynecologic Oncology* **136** (2015), 189–197.

[22] N. Vasiljević, D. Scibior-Bentkowska, A.R. Brentnall, J. Cuzick and A.T. Lorincz. Credentialing of DNA methylation assays for human genes as diagnostic biomarkers of cervical intraepithelial neoplasia in high-risk HPV positive women. *Gynecologic Oncology* **132** (2014), 709–714.

[23] M.S. Pepe, K.F. Kerr, G. Longton and Z. Wang (2013). Testing for improvement in prediction model performance. *Statist Med* **32** (9), 1467–1482.

### Supplementary data

Table S1

Univariate and multivariate tests of model components. LR-1 is the univariate likelihood-ratio $\chi_1^2$; LR-2 is the decrease when dropping that variable from the full model; LR-3 is the stepwise contribution in the order of the table so that *EPB41L3* is added first because it is common to all samples, then the HPV predictors are added starting with HPV16 L1. -Log10 P values are given in brackets, where -Log10 $P = 2, 3, 4$ if $P = 0.01, 0.001, 0.0001$ etc. The Spearman correlation coefficient of each HPV variable with *EPB41L3* is also provided. The overall likelihood ratio $\chi_7^2$ when fitting all terms was 453.86

| | LR-1 | LR-2 | LR-3 | *EPB41L3* Spearman |
|---|---|---|---|---|
| *EPB41L3* | 152.5 (34.3) | 72.4 (16.8) | 152.5 (34.3) | |
| HPV16 L1 | 214.2 (47.8) | 63.0 (14.7) | 174.7 (39.2) | 0.18 |
| HPV16 L2 | 190.6 (42.6) | 33.3 (8.1) | 26.2 (6.5) | 0.24 |
| HPV31 L1 | 31.4 (7.7) | 40.7 (9.7) | 35.6 (8.6) | 0.10 |
| HPV18 L2 | 14.1 (3.8) | 20.0 (5.1) | 17.8 (4.6) | 0.28 |
| HPV33 L1 | 41.3 (9.9) | 47.2 (11.2) | 47.2 (11.2) | 0.28 |

